# Supplementary material for: Selection for increased tibia length in mice alters skull shape through parallel changes in developmental mechanisms
Source: eLife. 2021 Apr 26;10:e67612. doi: 10.7554/eLife.67612 (PMC8118654; doi:10.7554/eLife.67612)
Supplement: Supplementary file 1. — Body mass data represent means and SEM, whereas centroid size and tibia length are least squared means and SEM. Superscripts denote significant differences in means (p<0.05) between a given group and: Controls CTL, Longshanks Line 1 LS1, Longshanks Line 2 LS2, from either: Generation 1 F01, Generation 9 F09, or Generation 20 F20, as determined using Tukey’s HSD tests. Differences in body mass were determined by ANOVA, whereas tibia length and centroid size differences were assessed by ANCOVA with body mass as a covariate. Bold and italic superscripts indicate significant intergenerational differences and intragenerational differences, respectively. [file elife-67612-supp1.docx]

Supplementary File 1 – Morphometric data for adult mice among lines and generations. Body mass data represent means and SEM, whereas centroid size and tibia length are least squared means and SEM. Superscripts denote significant differences in means (p < 0.05) between a given group and: Controls ^CTL^, Longshanks Line 1 ^LS1^, Longshanks Line 2 ^LS2^, from either: Generation 1 ^F01^, Generation 9 ^F09^, or Generation 20 ^F20^, as determined using Tukey’s HSD tests. Differences in body mass were determined by ANOVA, whereas tibia length and centroid size differences were assessed by ANCOVA with body mass as a covariate. Bold and italic superscripts indicate significant intergenerational differences and intragenerational differences, respectively.

| **Adult Body Mass (g)** | | | |
| --- | --- | --- | --- |
| Generation | CTL | LS1 | LS2 |
| F01 | 39.61 (1.28) | 39.25 (0.99) | 42.13 (0.99) **^F09 CTL, F09 LS2^** |
| F09 | 36.54 (1.30) **^F01 LS2^** | 35.91 (0.99) | 38.33 (0.99) **^F01 LS2^** |
| F20 | 40.05 (0.99) | 39.89 (0.99) | 39.81 (0.99) |
| **Adult Tibia Length (mm)** | | | |
| Generation | CTL | LS1 | LS2 |
| F01 | 18.21 (0.12) | 18.32 (0.09) **^F09-LS1, F20-LS1^** | 18.30 (0.09) **^F09-LS2, F20-LS2^** |
| F09 | 18.23 (0.12) *^F09-LS1, F09-LS2^* | 19.51 (0.09) **^F01-LS1, F20-LS1^**^,^ *^F09-CTL^* | 19.50 (0.09) **^F01-LS2, F20-LS2^**^,^ *^F09-CTL^* |
| F20 | 18.13 (0.09) *^F20-LS1, F20-LS2^* | 21.13 (0.09) **^F01-LS1, F09-LS1^**^,^ *^F20-CTL^* | 21.15 (0.09) **^F01-LS2, F09-LS2^**^,^ *^F20-CTL^* |
| **Adult Cranial Centroid Size** | | | |
| Generation | CTL | LS1 | LS2 |
| F01 | 56.66 (0.19) | 56.81 (0.15) **^F20-LS1^** | 56.51 (0.15) **^F20-LS2^** |
| F09 | 56.64 (0.19) | 57.49 (0.15) **^F20-LS1^** | 56.82 (0.15) **^F20-LS2^** |
| F20 | 56.64 (0.15) *^F20-LS1, F20-LS2^* | 58.07 (0.15) **^F01-LS1, F09-LS1^**^,^ *^F20-CTL^* | 57.81 (0.15) **^F01-LS2, F09-LS2^**^,^ *^F20-CTL^* |
