# Supplementary material for: Selection for increased tibia length in mice alters skull shape through parallel changes in developmental mechanisms
Source: eLife. 2021 Apr 26;10:e67612. doi: 10.7554/eLife.67612 (PMC8118654; doi:10.7554/eLife.67612)
Supplement: Supplementary file 2. — Euclidean distances between the multivariate mean PC scores of each group, based on Procrustes shape data adjusted for sex only (above diagonal), or sex and cranial centroid size (below diagonal). The only non-significant Euclidean distance, based on a post-hoc Procrustes ANCOVA, is indicated in bold. [file elife-67612-supp2.docx]

Supplementary File 2: Mean Euclidean distances. Euclidean distances between the multivariate mean PC scores of each group, based on Procrustes shape data adjusted for sex only (above diagonal), or sex and cranial centroid size (below diagonal). The only non-significant Euclidean distance, based on a post-hoc Procrustes ANCOVA, is indicated in bold.

|  | **Mean PC scores adjusted for sex only** | | | | | | | | | | |
| --- | --- | --- | --- | --- | --- | --- | --- | --- | --- | --- | --- |
| **Mean PC scores adjusted for centroid size and sex** |  | F01-CTL | F01-LS1 | F01-LS2 | F09-CTL | F09-LS1 | F09-LS2 | F20-CTL | F20-LS1 | F20-LS2 |  |
|  | F01-CTL | - | 0.010 | **0.007** | 0.012 | 0.013 | 0.015 | 0.010 | 0.022 | 0.023 |  |
|  | F01-LS1 | 0.011 | - | 0.011 | 0.014 | 0.016 | 0.018 | 0.014 | 0.025 | 0.026 |  |
|  | F01-LS2 | **0.007** | 0.011 | - | 0.012 | 0.011 | 0.013 | 0.009 | 0.020 | 0.020 |  |
|  | F09-CTL | 0.011 | 0.014 | 0.010 | - | 0.012 | 0.012 | 0.013 | 0.027 | 0.026 |  |
|  | F09-LS1 | 0.013 | 0.016 | 0.011 | 0.010 | - | 0.011 | 0.014 | 0.020 | 0.021 |  |
|  | F09-LS2 | 0.015 | 0.018 | 0.013 | 0.011 | 0.011 | - | 0.013 | 0.023 | 0.018 |  |
|  | F20-CTL | 0.010 | 0.014 | 0.010 | 0.012 | 0.014 | 0.013 | - | 0.021 | 0.019 |  |
|  | F20-LS1 | 0.016 | 0.020 | 0.015 | 0.018 | 0.015 | 0.018 | 0.015 | - | 0.015 |  |
|  | F20-LS2 | 0.018 | 0.022 | 0.016 | 0.019 | 0.018 | 0.013 | 0.014 | 0.015 | - |  |
