# Supplementary material for: Selection for increased tibia length in mice alters skull shape through parallel changes in developmental mechanisms
Source: eLife. 2021 Apr 26;10:e67612. doi: 10.7554/eLife.67612 (PMC8118654; doi:10.7554/eLife.67612)
Supplement: Supplementary file 3. — Tibia and centroid size data represent least squared means (SEM) and litter sizes are means (SEM). Differences in litter size were determined by ANOVA, whereas tibia length and centroid size differences were assessed by ANCOVA with litter size as a covariate. Superscripts denote significant differences in means (p<0.05) between a given group and: Controls CTL, Longshanks Line 1 LS1, Longshanks Line 2 LS2. [file elife-67612-supp3.docx]

Supplementary File 3 – Morphometric data for neonate mice among lines and generations. Tibia and centroid size data represent least squared means (SEM) and litter sizes are means (SEM). Differences in litter size were determined by ANOVA, whereas tibia length and centroid size differences were assessed by ANCOVA with litter size as a covariate. Superscripts denote significant differences in means (p < 0.05) between a given group and: Controls ^CTL^, Longshanks Line 1 ^LS1^, Longshanks Line 2 ^LS2^.

| **Neonate Morphometric Data** | | | |
| --- | --- | --- | --- |
| Line | CTL | LS1 | LS2 |
| Ossified Tibial Diaphysis Length (mm) | 6.66 (0.06) ^LS1, LS2^ | 7.50 (0.07) ^CTL^ | 7.41 (0.10) ^CTL^ |
| Litter Size | 7.94 (0.55) ^LS1, LS2^ | 10.33 (0.51) ^CTL^ | 11.83 (0.51) ^CTL^ |
| Centroid Size | 38.49 (0.17) ^LS1^ | 39.05 (0.16) ^CTL, LS2^ | 37.94 (0.16) ^LS1^ |
