# Supplementary material for: Selection for increased tibia length in mice alters skull shape through parallel changes in developmental mechanisms
Source: eLife. 2021 Apr 26;10:e67612. doi: 10.7554/eLife.67612 (PMC8118654; doi:10.7554/eLife.67612)
Supplement: Supplementary file 4. [file elife-67612-supp4.docx]

Supplementary File 4 – Adult cranium landmarks and their anatomical definition

| **Adult Cranium Landmarks** | |
| --- | --- |
| **Paired Landmarks (R/L)** | |
| Lateral point on frontal suture | 4/5 |
| Lateral zygomatic-frontal suture | 6/7 |
| Posterior zygomaticofrontal junction | 8/9 |
| Posterior margin of malar process | 10/11 |
| Frontal-temporal-parietal junction | 12/13 |
| Anterior margin of incisive foramen | 14/15 |
| Medial maxilla-premaxilla junction | 16/17 |
| Anterior inferior zygomatic | 18/19 |
| Anterior temporo-zygomatic junction | 20/21 |
| Anterior superior alveoli | 22/23 |
| Posterior incisive foramen | 24/25 |
| Point along palatine-maxillary suture | 26/27 |
| Medial palatal-pterygoid junction | 28/29 |
| Posterior superior alveoli | 30/31 |
| Lateral palatal-pterygoid junction | 32/33 |
| Spheno-occipital synchondrosis | 34/35 |
| Anterior foramen ovale | 36/37 |
| Posterior temporo-zygomatic junction | 38/39 |
| Auditory-temporal-sphenoid junction | 40/41 |
| Anterior inferior auditory bulla | 42/43 |
| Occipital-auditory-sphenoid junction | 44/45 |
| Point along occipitomastoid suture | 46/47 |
| Medial occipital condyle | 48/49 |
| Anterior nasal and premaxilla | 50/51 |
| Frontal suture on orbital rim | 52/53 |
| Superior temporo-zygomatic suture | 54/55 |
| Posterior zygomatic process | 56/57 |
| Superio-posterior tympanic ring | 58/59 |
| Occipital-auditory junction | 60/61 |
| Midline superior incisor | 62/63 |
| **Midline Landmarks** | |
| Lambda | 1 |
| Bregma | 2 |
| Nasion | 3 |
| Anterior foramen magnum | 64 |
| Midline junction basioccipital and sphenoid | 65 |
| Midline junction sphenoid and presphenoid | 66 |
| Anterior junction endocranial presphenoid | 67 |
| Endocranial junction frontal and ethmoid | 68 |
