# Supplementary material for: Selection for increased tibia length in mice alters skull shape through parallel changes in developmental mechanisms
Source: eLife. 2021 Apr 26;10:e67612. doi: 10.7554/eLife.67612 (PMC8118654; doi:10.7554/eLife.67612)
Supplement: Supplementary file 5. [file elife-67612-supp5.docx]

Supplementary File 5 – Neonate cranium landmarks and their anatomical definitions.

| **Neonate Cranium Landmarks** | |
| --- | --- |
| **Paired Landmarks (L/R)** | |
| Medial rostral tip of nasal bones | 1/2 |
| Caudal dorsal fronto-zygomatic suture on frontal bone | 4/5 |
| Rostral dorsal zygomatic suture on zygomatic bone | 6/7 |
| Caudal dorsal zygomatic suture on zygomatic process of temporal bone | 8/9 |
| Temporal-parietal-frontal suture | 10/11 |
| Caudal medial tip of frontal bones (bregma) | 12/13 |
| Ventral caudal tip of occipital process of temporal bone | 14/15 |
| Caudal medial tip of the parietal | 16/17 |
| Caudal lateral tip of interparietal | 18/19 |
| Rostral incisor foramen | 22/23 |
| Premaxilla-maxilla suture (rostral medial maxilla) | 24/25 |
| Maxilla-palatine suture (rostral medial palatine) | 26/27 |
| Caudal medial tip of palatine | 28/29 |
| Dorsal lateral tip of exoccipital | 30/31 |
| Medial border of exoccipital at widest mediolateral span of foramen magnum | 32/33 |
| Ventral tip of paraoccipital process | 34/35 |
| Caudal medial tip of basioccipital at intra-occipital synchondrosis | 36/37 |
| Caudal lateral tip of basioccipital at intra-occipital synchondrosis | 39/40 |
| Rostral lateral tip of basioccipital at spheno-occipital synchondrosis | 41/42 |
| Caudal lateral tip of sphenoid at spheno-occipital synchondrosis | 43/44 |
| Rostral lateral tip of sphenoid at intersphenoidal synchondrosis | 45/46 |
| Caudal lateral tip of presphenoid at intersphenoidal synchondrosis | 47/48 |
| **Midline Landmarks** | |
| Caudal medial nasal bones (nasion) | 3 |
| Caudal medial border of interparietal | 20 |
| Ventral medial occipital (dorsal foramen magnum) | 21 |
| Caudal medial border of basioccipital (rostral foramen magnum) | 38 |
| Ethmoid-presphenoid suture | 49 |
| Rostral medial border of cribriform plate | 50 |
