# Supplementary material for: Selection for increased tibia length in mice alters skull shape through parallel changes in developmental mechanisms
Source: eLife. 2021 Apr 26;10:e67612. doi: 10.7554/eLife.67612 (PMC8118654; doi:10.7554/eLife.67612)
Supplement: Supplementary file 6. [file elife-67612-supp6.docx]

| **Sagittal Midline Neonate Cranial Base Landmarks** | |
| --- | --- |
| Basion | 1 |
| Rostral dorsal tip of basi-occipital at spheno-occipital synchondrosis | 2 |
| Caudal dorsal tip of basi-sphenoid at spheno-occipital synchondrosis | 3 |
| Rostral dorsal tip of basi-sphenoid at intersphenoidal synchondrosis | 4 |
| Caudal dorsal tip of presphenoid at intersphenoidal synchondrosis | 5 |
| Ethmoid-presphenoid suture | 6 |
| Rostral medial border of cribriform plate | 7 |
| Rostral ventral tip of basi-occipital at spheno-occipital synchondrosis | 8 |
| Caudal ventral tip of basi-sphenoid at spheno-occipital synchondrosis | 9 |
| Rostral ventral tip of basi-sphenoid at intersphenoidal synchondrosis | 10 |
| Caudal ventral tip of presphenoid at intersphenoidal synchondrosis | 11 |
| Rostral ventral tip of the presphenoid | 12 |

Supplementary File 6 – Neonate cranial base landmarks and their anatomical definitions.
